# Petroleum and Chlorinated Solvents in Meconium and the Risk of Hypospadias: A Pilot Study

**DOI:** 10.3389/fped.2021.640064

**Published:** 2021-06-02

**Authors:** Florence Rouget, Adèle Bihannic, Sylvaine Cordier, Luc Multigner, Marie Meyer-Monath, Fabien Mercier, Patrick Pladys, Ronan Garlantezec

**Affiliations:** ^1^CHU Rennes, Univ Rennes, Inserm, EHESP, Irset (Institut de recherche en santé, environnement et travail)—UMR_S 1085, Rennes, France; ^2^Brittany Registry of Congenital Anomalies, CHU Rennes, Rennes, France; ^3^Univ Rennes, Inserm, EHESP, Irset (Institut de recherche en santé, environnement et travail)—UMR_S 1085, Rennes, France; ^4^INERIS, Verneuil-en-Halatte, France; ^5^LERES, EHESP, Irset (Institut de recherche en santé, environnement et travail)—UMR_S 1085, Rennes, France; ^6^Univ Rennes, CHU Rennes, Inserm, LTSI-UMR 1099, Rennes, France

**Keywords:** hypospadias, BTEX, petroleum solvents, chlorinated solvents, meconium, volatile organic compound

## Abstract

**Background:** Hypospadias is a male congenital malformation that occurs in ~2 of 1,000 births. The association between hypospadias and fetal exposure to environmental chemicals has been studied, but the results are inconsistent. Although several petroleum and chlorinated solvents are suspected to have teratogenic effects, their role in the occurrence of hypospadias has been little studied and never using biomarkers of exposure. We aimed to evaluate the association between fetal exposure to petroleum and chlorinated solvents measured in meconium and the occurrence of hypospadias.

**Methods:** We conducted a pilot case-control study in the maternity of the University Hospital of Rennes (France). Eleven cases of hypospadias and 46 controls were recruited between October 2012 and January 2014. Data from hospital records and maternal self-reported questionnaires, including socio-demographic characteristics and occupational and non-occupational exposure to chemicals, were collected. Meconium samples were collected using a standardized protocol. Levels of petroleum solvents (toluene, benzene, ethylbenzene, and p, m, and o xylene), certain metabolites (mandelic acid, hippuric acid, methylhippuric acid, S-phenylmercapturic acid, S-benzylmercapturic acid, and phenylglyoxylic acid), and two chlorinated solvents (trichloroethylene and tetrachloroethylene) were measured in meconium by gas and liquid chromatography, both coupled to tandem mass spectrometry. Associations between the concentration of each chemical and the occurrence of hypospadias were analyzed using exact logistic regressions adjusted for maternal age, educational level, pre-pregnancy body mass index, and alcohol, and tobacco consumption during pregnancy. Results are presented with odds ratios (ORs) and their 95% confidence intervals (CIs).

**Results:** Quantification rates for petroleum and chlorinated solvents or metabolites ranged from 2.2% (for methylhippuric acid) to 77.1% (for trichloroethylene) of the meconium samples. We found a significant association between the quantification of phenylglyoxylic acid (metabolite of styrene and ethylbenzene) in the meconium and a higher risk of hypospadias (OR = 14.2, 95% CI [2.5–138.7]). The risk of hypospadias was non-significantly elevated for most of the other solvents and metabolites.

**Conclusion:** This exploratory study, on a limited number of cases, suggests an association between petroleum solvents and hypospadias. Additional studies are needed to confirm these results and identify the determinants for the presence of these solvents in meconium.

## Introduction

Hypospadias is a male congenital malformation defined by a displaced urethral meatus on the ventral surface of the penis. The prevalence of hypospadias is ~2 in 1,000 total births (male and female) according to EUROCAT and the International Clearinghouse for Birth Defects and Surveillance and Research data (1, 2). However, the geographical variation of its prevalence is large, depending on how it is defined and ascertained, ranging from 0.2 to 3.9 per 1,000 births (2). The etiology remains largely unknown and is likely to be multifactorial, probably involving both genetic and environmental factors (3).

Among the most widespread chemicals, organic solvents are present in a large range of products used at home or at work, including paints, varnishes, inks, and cleaning agents and may be present as volatile organic compounds (VOCs) for some. There are three major organic solvent chemical families: oxygenated, petroleum, and chlorinated solvents. Their main routes of exposure are dermal and respiratory. Although several studies have suggested an association between *in utero* exposure to organic solvents and hypospadias (4–11), only a few have focused on the subtypes (7, 10, 11). The studies of Cordier et al. (10) and Warembourg et al. (11) suggested an association between the presence of metabolites of urinary glycol ethers, an oxygenated solvent chemical family, during pregnancy and hypospadias. Another study suggested a non-significant elevated risk of hypospadias associated with tetrachloroethylene-contaminated drinking water (estimated using water distribution system modeling software) (7). Certain petroleum solvents, such as “BTEX” (benzene, toluene, ethylbenzene, and *o,m,p*-xylene), are suspected to act as endocrine disruptors (12) that may affect the hormonal environment during pregnancy and thus the genital development of the fetus. However, they have never been studied in relation to the occurrence of hypospadias in newborns.

Exposure to chlorinated and petroleum solvents could be potentially assessed using biomarkers in urine or serum. However, their relatively short half-lives, especially that of petroleum solvents, limit the assessment of exposure throughout pregnancy, especially during the specific windows of susceptibility. Meconium is a useful matrix to assess fetal exposure (13, 14). It is the first feces to be expelled by the neonate after birth. It is composed of water, lipids, and proteins derived from swallowed amniotic fluid and secretions and desquamated epithelial cells from the fetal intestine (13). It starts being produced from around the 12th to 13th week of gestation, accumulates in the colon, and is passed by the neonate within the first 24–48 h after birth (14). It may therefore reflect cumulative fetal exposure, even to toxicants with a short half-life (parent molecules or metabolites). Its collection is non-invasive and simple and it provides unequivocal evidence that the chemical has reached the fetus (15). It has already been used to detect fetal exposure to various toxicants, such as illicit drugs (16), alcohol, certain pesticides (17–19), and various non-persistent organic pollutants (20).

The aim of this exploratory study was to evaluate the association between fetal petroleum and chlorinated solvent levels measured in meconium and the risk of hypospadias.

## Materials and Methods

### Population

We conducted a pilot case-control study on a subsample of the PENEW (Pregnancy Environment and NEWborn) project, led by the Brittany Registry of Congenital Anomalies, to study the impact of environmental chemical exposure (solvents and pesticides) on congenital malformations in Brittany (France) between October 2012 and December 2018. Cases in the PENEW project consisted of neonates or fetuses affected by any congenital malformation diagnosed at birth in one of the 13 participating maternity wards in Brittany. For each case, the controls were the two following livebirths of the same gender without any birth defects, born in the same maternity and not hospitalized in a neonatal unit. For both cases and controls, exclusion criteria were residence of the mother outside of Brittany and refusal to participate. All cases and controls were ascertained by a pediatrician in charge of the Brittany congenital malformation registry and trained according to EUROCAT classification guidelines (21).

For this study, we selected hypospadias cases and controls that were recruited at birth in the maternity ward of the University Hospital in Rennes during the first 15 months of enrolment (between October 7, 2012 and January 6, 2014).

During the inclusion period, ~5,000 livebirths were recorded in the maternity ward. Cases (*N* = 11) consisted of male live births diagnosed with hypospadias on the first day of life by a pediatrician. They represented 69% (11/16) of the eligible children born in the maternity during the same period and diagnosed with hypospadias within the first day of life (before expelling the meconium). Hypospadias was defined as an abnormal location of the urethral orifice on the ventral surface of the penis and coded using the International Classification Disease 10 (ICD 10), following EUROCAT guidelines (21). This classification is based on the location of the ectopic meatus, i.e., balanic (Q540), penile (Q541), penoscrotal (Q542), or perineal (Q543). Hypospadias associated with chromosomal and genetic conditions was excluded. All cases were confirmed by a pediatric surgeon and included in the Brittany registry of congenital anomalies.

Controls (*N* = 47) consisted of male live birth controls of all PENEW cases (excluding those of syndromic and chromosomic cases) with available meconium analysis (*N* = 46) and delivered during the same time period. This enabled us to obtain a ratio of one case to four controls to maximize the power of the study.

### Data Collection

During their stay in the maternity ward, the mothers completed a questionnaire after delivery that provided information on their medical history, tobacco exposure during pregnancy (maternal consumption and passive smoking), alcohol consumption, medication during pregnancy, food habits, and occupational and domestic exposure to a large number of environmental pollutants.

Medical data, including obstetrical history, the health of the newborn, and a description of the malformation (for cases), came from hospital medical records, including clinical examination by the pediatrician before discharge and follow-up by pediatric surgeons.

### Indirect Assessment of Exposure to Solvents

We assessed maternal occupational exposure to solvents during pregnancy using two job-exposure matrices (JEMs) constructed by Santé Publique France (French national public health agency), one concerning petroleum solvents (22) and the other concerning chlorinated solvents (23). Each allowed the assessment of exposure to the family of solvents in a single group (petroleum and chlorinated solvents) and to subgroups of solvents within each family: benzene, other aliphatic mineral spirits, motor gasoline, white-spirits, and other light aromatic mixtures, gasoil, fuels, and kerosene for petroleum solvents and chloroform, methylene chloride, tetrachloroethylene, trichloroethylene, and 1,1,1-trichloroethane for chlorinated solvents.

In addition, information about the domestic use of products containing solvents during pregnancy was collected from the questionnaire: frequency of damp cleaning (<5 times/month, between 5 and 20 times/month, more than 20 times/month), home renovation activities (yes/no), and other chemicals used during leisure activities (yes/no) during pregnancy.

### Meconium Collection

Three meconium samples were collected into dedicated stool-culture containers by healthcare staff within the first 2 days of life following a standardized procedure for both cases and controls. Only water and cotton pads were used to clean the diaper area, excluding any use of cream or lotions. The healthcare staff was instructed to use gloves (without talc) instead of hydroalcoholic gel during collection to prevent any contamination of the samples. Samples were immediately frozen and initially stored in a dedicated freezer at −20°C, in the maternity ward, and then transferred to The French National Institute for Industrial Environment and Risk (INERIS) laboratory and stored at −80°C until analysis.

Possible contamination of the diapers by BTEX or chlorinated solvents that could have led to the contamination of the meconium samples was assessed by analyzing the three references of diapers used in the maternity ward during the study period. Details of the chemical analysis, including quality assurance and quality control (QA/QC), are provided in Supplementary Material 1. Under these conditions, no BTEX or chlorinated solvents were quantifiable in the three references, except toluene, which was detected at slightly above the limit of quantification for only one.

### Chemical Analysis of Meconium Samples

Meconium samples were analyzed by the INERIS laboratory. A specific analytic method based on headspace (HS) solid phase microextraction (SPME) gas chromatography coupled with mass spectrometry (GC/MS) was developed to detect and quantify aromatic hydrocarbons “BTEX” (benzene, toluene, ethylbenzene, and *o,m,p*-xylene) and two chlorinated solvents (trichloroethylene and tetrachloroethylene). This method has been described elsewhere (24). The limits of quantification (LOQ) ranged from 0.08 ng/g of meconium (tetrachloroethylene) to 0.12 ng/g (benzene, toluene) (24). Metabolites of BTEX [o-cresol, hippuric acid, mandelic acid (MA), 2-methylhippuric acid (MHA), S-phenylmercapturic acid (s-PMA), and benzylmercapturic acid (s-BMA)], and one metabolite of styrene and ethylbenzene [phenylglyoxylic acid (PGA)] were analyzed by liquid chromatography coupled to tandem mass spectrometry (LC/MS/MS) according to a previously described method (25). The limits of quantification ranged from 4.8 ng/g (MHA) to 174.7 ng/g (o-cresol) (25). Analyses were blindly performed for cases and controls.

### Statistical Analysis

Concentrations of chemicals were classified into quartiles. For association analyses, two categorizations were considered with different cut-offs according to the frequency of quantification among controls. The cut-off was the third quartile if the quantification frequency was above 50% (i.e., for toluene, benzene, and trichloroethylene) or the limit of quantification if the quantification frequency was below 50% (i.e., for other solvents).

Correlations between solvents and metabolites were studied using chi-square or Fisher exact tests, Wilcoxon tests, or Spearman rank correlations, depending on the level of quantification. We explored the relationship between solvent or metabolite concentrations and certain potential sources: tobacco consumption during pregnancy, occupational exposure to chlorinated and petroleum solvents, self-reported domestic uses of products that may contain solvents (for damp cleaning, home renovation, and leisure activities) using chi-square or Fisher exact tests.

We used an exact logistic regression model to study the risk of hypospadias related to BTEX or chlorinated solvents to reduce the small sample bias, which generally leads to overestimation of the true value (26). Associations were tested independently for each chemical and only for those detected in more than 10% of samples. Potential confounders included in the adjustment set of variables were chosen a priori: maternal age (<25, 25–35, ≥35 years), education level (high school or less, post-secondary school), body-mass index (<25, ≥25 m/kg^2^), tobacco consumption during pregnancy (yes/no), and alcohol consumption during pregnancy (yes/no). Results are presented as odds ratios (ORs) with their 95% confidence intervals (CIs).

We did not correct *p*-values for multiple testing and accepted the possibility of false discoveries, as our study was a pilot study. Nevertheless, and, as recommended (27, 28), all comparisons were planned a priori and all results are reported.

All statistical analyses were performed using SAS statistical software (version 9.4, SAS Institute Inc., Cary, NC, USA) and LogXact 10 (Cytel).

## Results

The characteristics of the cases and controls are presented in Table 1. Mothers of cases were significantly less educated and more affected by hypertension than those of controls. There were no other significant differences between cases and controls in terms of other maternal characteristics or environmental exposure.

**Table 1:** Characteristics of the 11 cases of hypospadias and 46 controls.

		**Controls (*****n*** **= 46)**		**Cases (*****n*** **= 11)**		***p-*value**
		***n***	**%**	***n***	**%**	
Age	<25	3	6.5	1	9.1	0.91
	25–35	37	80.4	9	81.8	
	≥35	6	13.0	1	9.1	
	mean (± SD)	30.2 (±3.6)		29.3 (± 4.2)		0.62
Educational level	High school or less	10	21.7	7	63.6	0.006
	Postsecondary school	36	78.3	4	36.7	
Work during pregnancy	No	29	63.0	6	55.5	0.73
	Yes	17	37.0	5	45.5	
BMI (kg/m^2^)	<25	33	71.7	7	63.6	0.60
	≥25	13	28.3	4	36.4	
	mean (± SD)	23.5 (± 4.2)		24.3 (± 5.0)		0.66
Tobacco consumption during pregnancy	No	31	67.4	7	63.6	0.81
	Yes	15	32.6	4	36.4	
Passive smoking during pregnancy	No	16	38.1	4	36.4	0.92
	Yes	26	61.9	7	63.6	
	missing	4	–	0		
Alcohol consumption during pregnancy	No	40	87.0	11	100.0	0.20
	Yes	6	13.0	0	0.0	
Vitamin Supplementation (beginning pregnancy)	No	25	55.6	6	60.0	0.80
	Yes	20	44.4	4	40.0	
	missing	1	–	1	–	
Diabetes during pregnancy	No	44	95.7	10	90.9	0.53
	Yes	2	4.3	1	9.1	
Hypertension (before and during pregnancy)	No	46	100.0	8	72.7	0.003
	Yes	0	0.0	3	27.3	
SGA 10th percentile (AUDIPOG curves)	No	38	82.6	8	72.7	0.43
	Yes	8	17.4	3	27.3	

*BMI, body-mass index; SGA, small for gestational age*.

Among the 11 cases of hypospadias, four were balanic (Q540), five penile (Q541), and two penoscrotal (Q542). Only penoscrotal cases were associated with another malformation (one of esophageal atresia and one of multicystic renal dysplasia).

The frequency of quantification and distribution (in quartiles) of chemical concentrations in the meconium of controls are presented in Table 2. Solvents were quantified from 2.2% of samples for MHA and S-PMA to 63.9% for benzene and 77.1% for trichloroethylene.

**Table 2:** Distribution of chemical concentrations in meconium samples (BTEX and chlorinated solvents and metabolites) among controls (*n* = 46).

	***N***	**LOQ (ng/g)**	***N* (%)**	**P25 (ng/g)**	**P50 (ng/g)**	**P75 (ng/g)**	**Maximum (ng/g)**
			**>LOQ**	**Q1**	**Q2**	**Q3**	
Toluene	35	0.120	22 (62.9)	<LOQ	3.78	94.9	538
Benzene	46	0.120	33 (63.9)	<LOQ	0.300	1.07	41.0
Ethylbenzene	46	0.120	20 (43.5)	<LOQ	<LOQ	0.540	3.17
p-xylene	46	0.100	14 (30.5)	<LOQ	<LOQ	0.120	0.920
m-xylene	46	0.120	15 (32.6)	<LOQ	<LOQ	0.320	1.67
o-xylene	46	0.120	6 (13.0)	<LOQ	<LOQ	<LOQ	2.54
Phenylglyoxylic acid (PGA)	46	29.9	3 (6.5)	<LOQ	<LOQ	<LOQ	403
O_cresol	46	175	6 (13.0)	<LOQ	<LOQ	<LOQ	960
Mandelic acid	46	72.2	5 (9.8)	<LOQ	<LOQ	<LOQ	122
Hippuric acid	46	17.4	17 (37.0)	<LOQ	<LOQ	29.9	1330
Methylhippuric acid (MHA)	46	4.82	1 (2.2)	<LOQ	<LOQ	<LOQ	113
S-phenylmercapturic acid (S-PMA)	46	51.6	1(2.2)	<LOQ	<LOQ	<LOQ	87.3
S-benzylmercapturic acid (S-BMA)	46	10.4	3 (6.5)	<LOQ	<LOQ	<LOQ	20.2
Trichloroethylene	46	0.100	34 (77.1)	<LOQ	0.210	0.350	0.900
Tetrachloroethylene	46	0.0800	22 (47.8)	<LOQ	<LOQ	0.230	0.380

*LOQ, limit of quantification*.

*Q1, 1st quartile; Q2, 2nd quartile; Q3, 3rd quartile*.

There were strong correlations between the levels of several solvents and metabolites (Supplementary Material 2). Such correlations were expected, as these solvents share common major sources of exposure, such as indoor and outdoor air pollution for petroleum solvents.

Among the mothers who declared working during pregnancy, only one was classified as potentially exposed at work to chlorinated solvents (probably to chloroform but not trichloroethylene or tetrachloroethylene) and another was classified as potentially exposed at work to petroleum solvents by the JEMs (probably to white spirit but not benzene). Hippuric acid was the only detected metabolite associated with smoking during pregnancy (Supplementary Material 2, Table 1). In terms of self-reported non-occupational exposure, the frequency of damp cleaning was associated with higher quantification rates of ethylbenzene, p-xylene, and o-xylene. Home renovation was associated with higher quantification rates of m-xylene, hippuric acid, and trichloroethylene (Supplementary Material 2, Table 1). Other self-reported exposure to chemicals during leisure-time activities was not associated with any changes in the levels of solvents or their metabolites measured in the meconium samples (Supplementary Material 2, Table 1).

The associations between the levels of chemical exposure and the risk of hypospadias are presented in Table 3. We found a significant association between the levels above the limit of quantification for PGA in meconium samples and a higher risk of hypospadias (OR = 14.2, 95% CI [2.5–138.7]). The risk of hypospadias was elevated for most of the other compounds and metabolites but was not statistically significant.

**Table 3:** Association between the levels of BTEX and chlorinated solvents and their metabolites in the meconium and the occurrence of hypospadias (*n* = 11 cases and 46 controls).

		**Cases (*N* = 11)**	**Controls (*N* = 46)**	**OR [95% CI]**	OR^*^ [95% CI]
Toluene^#^	<Q3	5	26	Ref	Ref
	≥Q3	2	9	0.87 [0.11–10.64]	2.91 [0.12–202.10]
Benzene	<Q3	8	34	Ref	Ref
	≥Q3	3	12	0.94 [0.18–6.41]	2.67 [0.28–34.34]
Ethylbenzene	<LOQ	4	26	Ref	Ref
	≥LOQ	7	20	2.27 [0.58–8.86]	1.81 [0.46–7.61]
p-Xylene	<LOQ	6	32	Ref	Ref
	≥LOQ	5	14	1.90 [0.50–7.29]	2.38 [0.53–11.17]
m-Xylene	<LOQ	4	31	Ref	Ref
	≥LOQ	7	15	3.61 [0.92–14.30]	3.17 [0.76–14.00]
o-Xylene	<LOQ	8	40	Ref	Ref
	≥LOQ	3	6	2.50 [0.51–12.39]	2.77 [0.43–17.55]
Phenylglyoxylic acid (PGA)	<LOQ	6	43	Ref	Ref
	≥LOQ	5	3	11.94 [2.25–63.25]	14.17 [2.45–138.66]
o-Cresol	<LOQ	9	40	Ref	Ref
	≥LOQ	2	6	1.48 [0.26–8.58]	2.12 [0.31–12.48]
Mandelic acid	<LOQ	11	41	Ref	Ref
	≥LOQ	0	5	–	–
Hippuric Acid	<LOQ	7	29	Ref	Ref
	≥LOQ	4	17	0.97 [0.24–3.82]	2.25 [0.42–14.57]
Methylhippuric acid (MHA)	<LOQ	11	45	Ref	Ref
	≥LOQ	0	1	–	–
S-phenylmercapturic acid (S-PMA)	<LOQ	11	45	Ref	Ref
	≥LOQ	0	1	–	–
S-benzylmercapturic acid (S-BMA)	<LOQ	10	43	Ref	Ref
	≥LOQ	1	3	0.70 [0.05–40.25]	0.62 [0.03–12.56]
Trichloroethylene	<Q3	7	34	Ref	Ref
	≥Q3	4	12	0.62 [0.13–3.43]	1.41 [0.22–8.04]
Tetrachloroethylene	<LOQ	6	24	Ref	Ref
	≥LOQ	5	22	0.91 [0.24–3.40]	1.25 [0.32–5.04]

# *Toluene measurements were available for seven cases and 35 controls*.

*OR, Odds ratio; 95% CI, 95% confidence interval*.

* *Adjusted for maternal age, educational level, body-mass index, and alcohol and tobacco consumption during pregnancy*.

*LOQ, limit of quantification (LOQ = 0.12 mg/g for ethylbenzene, m-xylene, o-xylene; 0.10 mg/g for p-xylene; 29.92 mg/g for PGA; 174.69 mg/g for O-cresol; 17.36 mg/g for hippuric acid; and 0.08 mg/g for tetrachloroethylene)*.

*Q3, third quartile (Q3 = 91.9 ng/g for toluene, 1.07 ng/g for benzene, and 0.35 ng/g for trichloroethylene)*.

## Discussion

This pilot study shows that several petroleum and chlorinated solvents and their metabolites can be quantified in meconium samples and suggests an association between quantification of PGA in meconium samples and a higher risk of hypospadias. The risk of hypospadias was elevated for most of the other detected solvents and metabolites but was not statistically significant.

This study had several strengths, including the direct assessment of fetal exposure via the analysis of biomarkers in meconium samples, which reflects cumulative exposure starting from the 12th to 13th week of gestation until birth (13, 14, 29). Interestingly, the cumulative exposure traced in this matrix covered the window of susceptibility for the development of hypospadias: human male urethral development starts from the indifferent stage of genital development at 6–8 weeks gestation, with progression of the urethral meatus from the scrotal folds to its terminal position on the glans, ending at 16–17 weeks (30, 31). The analytical methods were specifically developed and validated for this study (24, 25). Other strengths of our study were the ascertainment and validation of cases by pediatricians and surgeons, which avoided a misclassification bias, and a large number of potential confounders. Although maternal hypertension was associated with hypospadias, we decided a priori to not adjust for it, as it can be considered as an intermediate factor.

This study also had several limitations, mainly due to the small number of cases, leading to imprecision in the estimates (i.e., wide 95% confidence intervals). The low number of cases was due to the necessity of early inclusion after birth, and therefore the early diagnosis of hypospadias, to collect meconium before its being expelled. Certain forms of balanic hypospadias are hidden by an overlapping or tight prepuce and can be difficult to diagnose at birth; the diagnosis is sometimes made later during the stay at the maternity ward by the pediatrician, or even after discharge, by a surgeon. This constraint may have led to the selection of major forms. However, the repartition of anatomical subtypes of hypospadias (balanic, penile, and penoscrotal) was no different between the included and non-included cases born in the same maternity ward during the same period.

This pilot study is the first to focus on “BTEX-Styrene” (BTEX-S) or chlorinated solvent exposure in relation to the occurrence of hypospadias based on measurements in meconium. We found that petroleum solvents could be quantified in 15% to 65% of samples and most of their metabolites in <20%, except for hippuric acid. The levels observed in the present study are difficult to compare, as, to our knowledge, no other study has measured these compounds in meconium. Thus, comparison with other biomonitoring studies using other matrices are difficult to interpret, even if BTEX-S have been widely detected in the general population in blood samples (32) and their metabolites in blood and urine samples (33–35). For example, NHANES biomonitoring studies of urine among children in 2005–2012 (35) detected PGA in more than 90% of samples, suggesting that exposure to PGA is indeed widespread compared to the 6.5% of quantification in our study. However, little is known about the fetal pharmacokinetics and metabolism of PGA and we cannot currently infer external exposure associated with the potential increased risk suggested by our results. In non-occupational settings, the major source of exposure to BTEX-S comes from exposure to tobacco (35, 36), as well as indoor and outdoor air pollution (37–39). In addition, BTEX-S have been reported to be present in food (40, 41), although this source of exposure contributed less than other sources, except for styrene, for which food appeared to contribute to the same extent as airborne exposure (42). In our study, the levels of BTEX, their metabolites, and chlorinated solvents were not associated with occupational exposure or to tobacco (maternal consumption or passive exposure), except for hippuric acid (for maternal consumption only). The relationship between the presence of BTEX or their metabolites in the meconium and airborne or food sources could not be investigated in our study. However, the domestic use of solvents through cleaning habits (damp cleaning) or renovation activities was associated with more frequent detection of ethylbenzene, p-xylene, and o-xylene (damp cleaning), and m-xylene, hippuric acid, and trichloroethylene (home renovation). Home renovation has already been found to be associated with BTEX-S levels in a study on predictors of indoor BTEX concentrations in Canadian homes (43). Finally, although the French Agency for Food, Environmental and Occupational Health and Safety (ANSES) has reported that several brands of diapers contain BTEX-S, such as o-, m-, and p-xylene, toluene, and styrene (44), the analyses we conducted on the diapers used in our maternity ward before the study did not show any contamination by BTEX-S. We consequently did not consider the diapers themselves to be a possible source of these compounds. PGA, the only metabolite significantly associated with a higher risk of hypospadias in our study, has no known sources other than the metabolization of styrene and ethylbenzene (45).

Our study is the first to focus on the potential association of maternal exposure to BTEX and chlorinated solvents and the risk of hypospadias using objective measures for fetal assessment. Animal data have suggested that BTEX (46–48), styrene (49, 50), and chlorinated solvents (51, 52) can be teratogens. However, their association with male genital malformations has been rarely investigated. Although Bolden et al. suggested that BTEX-S may have endocrine disrupting properties at exposure levels below reference concentrations, the evidence is scarce (12, 52, 53). However, an experimental study in rats showed that styrene, one of the principal sources of PGA, has estrogenic properties that can affect genital development of the offspring, such as shortened anogenital distance, lower testis weight, and disruption of hormonal levels (41). In addition, ethylbenzene, the other precursor of PGA, was found to be associated with urinary tract malformations in the mouse in one study, but this was not observed in other developmental studies (54).

Our pilot study reports the feasibility of measuring petroleum solvents and chlorinated solvents and their metabolites in the meconium and suggests an association between petroleum solvents, especially PGA, and the occurrence of hypospadias. Although such an association is biologically plausible, we recognize that it may be due to a marker of co-exposure not investigated in our study, residual confounding or simply a chance finding. Given the small sample size, these results must be interpreted with caution and additional studies are needed to replicate them. If confirmed, these results may be of particular importance for the prevention of hypospadias.

## Data Availability Statement

The datasets presented in this article are not readily available because of regulatory restrictions. Requests to access the datasets should be directed to Violaine Benoit, violaine.benoit@chu-rennes.fr.

## Ethics Statement

The studies involving human participants were reviewed and approved by all required ethics committee in France: Person Protection Committee (Comité de Protection des Personnes Ouest V): reference 11/22-811 (06/09/2011); ANSM (AFSSAPS): ID-RCB: 2010—A01445-34—Ref. AFSSAPS: B110827-40—Authorization 01/07/2011; CCTIRS: n° 11.590—Approval on 20/11/2011; CNIL: n° 911526—Approval DR-2012-332 on 03/07/2012. Written informed consent to participate in this study was provided by the participants' legal guardian/next of kin and the mothers.

## Author Contributions

FR, SC, PP, LM, and RG contributed conception and design of the study. FR contributed to acquisition and interpretation of data. AB organized the database and contributed to the statistical analyses. MM-M performed the chemical analysis. RG performed the statistical analysis. FR and RG wrote the first draft of the manuscript. FR, RG, and FM wrote sections of the manuscript. All authors contributed to manuscript revision, read, and approved the submitted version.

## Conflict of Interest

The authors declare that the research was conducted in the absence of any commercial or financial relationships that could be construed as a potential conflict of interest.
