# Supplementary material for: Petroleum and Chlorinated Solvents in Meconium and the Risk of Hypospadias: A Pilot Study
Source: Front Pediatr. 2021 Jun 2;9:640064. doi: 10.3389/fped.2021.640064 (PMC8206475; doi:10.3389/fped.2021.640064)
Supplement: Supplementary file 1 [file Data_Sheet_1.pdf]

Supplementary material 1: Analysis of the diapers

Three different references of diapers provided by the University Hospital of Rennes were analyzed by the Environment and Health Research Laboratory (LERES) of the French School of Public Health (EHESP) to detect a possible contamination of the diapers by BTEX or chlorinated solvents that could have led to the contamination of the meconium samples.

Methodology of chemical analysis

Reagents and chemicals

ULC/MS-grade absolute methanol (MeOH) was purchased from Biosolve Chimie SARL (Dieuze, France). The standard 1,2-dibromoethane (internal standard (ISTD) for chlorinated solvents) was purchased from Merck KGaA (Darmstadt, Germany). The certified standard for benzene D6 (ISTD for BTEX) was purchased from LGC Labor GmbH (Augsburg, Germany). The purity of the standards was > 99%. Individual standard stock solutions of 1,2-dibromoethane and benzene D6 (2 g/L) were prepared in MeOH. The final multiple standard solution containing the two ISTDs was prepared in MeOH by the appropriate dilution of the individual standard stock solutions. A commercial MeOH mixture (5 g/L) of benzene, ethylbenzene, tetrachloroethylene, toluene, trichloroethylene, and m-, p-, and o-xylene was supplied by CARLO ERBA Reagents S.A.S (Val de Reuil, France). Calibration solutions were prepared by the appropriate dilution of this MeOH mixture into 100 mL mineral water (Evian) in 100-mL volumetric flasks and stored at 5°C for a maximum of 24 hours.

Sample preparation

Five milliliters of each calibration solution was pipetted into 20-mL headspace (HS) glass vials, which were then immediately sealed with an aluminum capsule with a butyl/polytetrafluoroethylene (PTFE) septum. Approximately 2 cm^2 of each diaper was cut at the place where the meconium was collected and placed into a 20-mL HS glass vial. Five milliliters of mineral water (Evian) was added and the vial was immediately sealed with an aluminum capsule with a butyl/PTFE septum. Five microliters of the ISTD solution was then introduced through the septum of all the HS glass vials.

Sample analysis

HS glass vials were placed in the 7697A Headspace Sampler (Agilent Technologies) for automated heating for 30 min at 60°C to ensure equilibration between the gas phase and the sample. An aliquot of the gas phase above the sample (1 mL) was automatically injected via a transfer line heated to 95°C into the GC split/splitless inlet heated to 150°C, operated in the split mode (split ratio: 10:1) and equipped with a 4.0-mm inner diameter (ID) low-pressure drop-inlet glass liner with deactivated wool (single taper, 6.3 mm OD × 78.5 mm × 4 mm ID).

The headspace sampler was interfaced with a 6890 GC system coupled to a 5973N mass selective detector (MSD) (Agilent Technologies) operated in the electron impact ionization (EI) mode (70 eV). Helium was used as column carrier gas at a constant flow rate of 0.9 mL/min. Chromatographic separation was performed on an Agilent J&W DB-624 capillary column (30 m length × 0.25 mm I.D., 1.4 µm film thickness), supplied by Agilent Technologies, with the following oven temperature program: 40°C (hold 5 min), first ramp at 5°C/min to 140°C (hold 1 min), and a second ramp at 20°C/min to 200°C (hold 0 min) to reach an analysis time of 29 min. The MSD transfer line, ion source, and quadrupole temperatures were fixed at 260, 230, and 150°C, respectively. The mass spectrometer (single quadrupole) was operated in the selected ion monitoring (SIM) mode. The two most intense

and specific ions of each compound were monitored for identification, confirmation, and quantification.

Quality assurance (QA) and quality control (QC)

All compounds were quantified from linear or quadratic calibration curves generated for each by analyzing at least four different calibration samples (from 0.25 to 20 µg/L for BTEX and 0.5 to 70 µg/L for chlorinated solvents). Each batch included: i) one procedural blank sample (5 mL mineral water) prepared and analyzed as a regular sample to assess whether the samples may have been contaminated during the analysis, ii) one calibration sample analyzed for every 20 samples to check the stability of the detector response, and iii) one calibration sample prepared from commercial solutions provided by other suppliers to validate the preparation of the calibration solutions.

Positive values for each substance were confirmed by comparing retention times between the calibration samples and the experimental samples. The data validation protocol of the method required that several conditions be met, including: i) the area of the ISTDs in a sample had to be within ± 25% of its area in the calibration samples, ii) the determination coefficient of the calibration curve had to be greater than 0.99, iii) the concentration of a substance in the procedural blank sample had to be lower than 50% of the LOQ, iv) the concentration of a substance in the calibration samples analyzed for every 20 samples had to be within ± 20% of its nominal concentration value, v) the concentration of a substance in the calibration sample at the LOQ level had to be within ± 50% of its nominal concentration value, and vi) the concentration of a substance in the calibration sample prepared from commercial solutions provided by other suppliers had to be within ± 20% of its nominal concentration value. If these conditions were not met, samples were re-analyzed if possible. Multiple precautions were taken to minimize procedural blank contamination. Plastic materials were avoided, glass materials were rigorously water rinsed prior to use, and samples were prepared in a room reserved for this purpose. Despite these precautions, BTEX compounds were detected in the procedural blank samples but were still far below the limits of quantification.

Results

Table 1. Amount of BTEX and trichloroethylene and tetrachloroethylene for 2 cm² of diaper (in ng).

	Benzene	Toluene	Ethylbenzene	m+p-Xylene	o-Xylene	Trichloro-ethylene	Tetrachloro-ethylene
LOQ	1.25	1.25	1.25	2.5	1.25	2.5	2.5
Diaper #1	< LOQ	1.55	< LOQ	< LOQ	< LOQ	< LOQ	< LOQ
Diaper #2	< LOQ	< LOQ	< LOQ	< LOQ	< LOQ	< LOQ	< LOQ
Diaper #3	< LOQ	< LOQ	< LOQ	< LOQ	< LOQ	< LOQ	< LOQ

LOQ: Limit of quantification

Conclusion

No BTEX or chlorinated solvents were quantifiable in the three references under these conditions, except toluene, which was detected slightly above the limit of quantification for only one reference.
