# Supplementary material for: Petroleum and Chlorinated Solvents in Meconium and the Risk of Hypospadias: A Pilot Study
Source: Front Pediatr. 2021 Jun 2;9:640064. doi: 10.3389/fped.2021.640064 (PMC8206475; doi:10.3389/fped.2021.640064)
Supplement: Supplementary file 2 [file Data_Sheet_2.pdf]

Supplementary Material 2

Associations between solvents and metabolites were measured with Chi square or Fisher's exact tests, Wilcoxon Test or Spearman correlation rank, depending on the level of quantification (i.e. below < 50% or ≥ 50%).

We observed several strong associations between solvents and their metabolites:

- toluene levels were positively correlated with quantification of ethylbenzene, p-xylene, o-xylene, m-xylene, and tetrachloroethylene;
- benzene levels were positively associated with quantification of ethylbenzene and tetrachloroethylene;
- ethylbenzene quantification was associated with higher quantification of p-xylene, o-xylene, m-xylene, tetrachloroethylene and levels of trichloroethylene;
- p-xylene quantification was associated with those of o-xylene and m-xylene;
- m-xylene quantification was associated with those of o-xylene ;
- o-xylene quantification was associated with trichloroethylene levels ;
- PGA quantification was associated with higher quantification of o-cresol ;
- o-cresol quantification was associated with higher quantification of hippuric acid and mandelic acid ;
- trichloroethylene and tetrachloroethylene levels were positively associated.

These numerous strong associations between petroleum solvents and their metabolites are in accordance with the common sources reported in the literature especially for BTEX and indoor and outdoor air pollution (1,2).

References:

1. Mohamed MF, Kang D, Aneja VP. Volatile organic compounds in some urban locations in United States. Chemosphere. juin 2002;47(8):863- 82.

2. Symanski E, Stock TH, Tee PG, Chan W. Demographic, residential, and behavioral determinants of elevated exposures to benzene, toluene, ethylbenzene, and xylenes among the U.S. population: results from 1999-2000 NHANES. J Toxicol Environ Health A. 2009;72(14):915-24.

Supplemental material 2, Table 1. Association between petroleum and chlorinated solvents and tobacco exposure during pregnancy and non-occupational chemical exposure.

	EthylBenzene					p-xylene					m-xylene				
	< LOQ		≥ LOQ			< LOQ		≥ LOQ			< LOQ		≥ LOQ		
	N	(%)	N	(%)	p-value	N	(%)	N	(%)	p-value	N	(%)	N	(%)	p-value
**Tobacco consumption during pregnancy**															
No	17	(56.7)	21	(77.8)		22	(57.9)	16	(84.2)		21	(60.0)	17	(77.3)	
Yes	13	(43.3)	6	(22.2)	0.16	16	(42.1)	3	(15.8)	0.07	14	(40.0)	5	(22.7)	0.25
**Passive smoking**															
No	12	(41.4)	8	(33.3)		16	(44.4)	4	(23.5)		12	(36.4)	8	(40.0)	
Yes	17	(58.6)	16	(66.7)	0.55	20	(55.5)	13	(76.5)	0.14	21	(63.6)	12	(60.0)	
missing	1	-	3	-		2	-	2	-		2	-	2	-	0.79
**Damp cleaning**															
< 5 times/month	25	(83.3)	14	(51.9)		30	(78.9)	9	(47.4)		26	(74.3)	13	(59.1)	
between 5 and 20 times/month	3	(10.0)	11	(40.7)		6	(15.8)	8	(42.1)		7	(20.0)	7	(31.8)	
> 20 times/month	2	(6.7)	2	(7.4)	0.01	2	(5.3)	2	(10.5)	0.04	2	(5.7)	2	(9.1)	0.47
**Home renovation activities**															
No	28	(93.3)	24	(88.9)		36	(94.7)	16	(84.2)		34	(97.1)	18	(81.8)	
Yes	2	(6.7)	3	(11.1)	0.67	2	(5.3)	3	(15.8)	0.32	1	(2.9)	4	(18.2)	0.05
**Other chemicals used during leisure activities**															
No	29	(96.7)	26	(96.3)		36	(94.7)	19	(100.0)		33	(94.3)	22	(100.0)	
Yes	1	(3.3)	1	(3.7)	0.95	2	(5.3)	0	(0.0)	0.55	2	(5.7)	0	(0.0)	0.52

Supplemental material 2, Table 1. Association between petroleum and chlorinated solvents and tobacco exposure during pregnancy and non-occupational chemical exposure.

	o-xylene					PGA					o-cresol				
	< LOQ		≥ LOQ			< LOQ		≥ LOQ			< LOQ		≥ LOQ		
	N	(%)	N	(%)	p-value	N	(%)	N	(%)	p-value	N	(%)	N	(%)	p-value
**Tobacco consumption during pregnancy**															
No	30	(62.5)	8	(88.9)		31	(63.3)	7	(87.5)		32	(65.3)	6	(75.0)	
Yes	18	(37.5)	1	(11.1)	0.25	18	(36.7)	1	(12.5)	0.25	17	(34.7)	2	(25.0)	0.71
**Passive smoking**															
No	19	(42.2)	1	(12.5)		17	(37.8)	3	(37.5)		17	(36.2)	3	(50.0)	
Yes	26	(57.8)	7	(87.5)	0.23	28	(62.2)	5	(62.5)	1.00	30	(63.8)	3	(50.0)	0.66
missing	3	-	1	-		4	-	0	-		2	-	2	-	
**Damp cleaning**															
< 5 times/month	36	(75.0)	3	(33.3)		33	(67.3)	6	(75.0)		33	(67.3)	6	(75.0)	
between 5 and 20 times/month	10	(20.8)	4	(44.4)		13	(26.5)	1	(12.5)		13	(26.5)	1	(12.5)	
> 20 times/month	2	(4.2)	2	(22.2)	0.02	3	(6.1)	1	(12.5)	0.44	3	(6.1)	1	(12.5)	0.43
**Home renovation activities**															
No	43	(89.6)	9	(100.0)		44	(89.8)	8	(100.0)		44	(89.8)	8	(100.0)	
Yes	5	(10.4)	0	(0.0)	0.59	5	(10.2)	0	(0.0)	0.91	5	(10.2)	0	(0.0)	0.9
**Other chemicals used during leisure activities**															
No	46	(95.8)	9	(100.0)		47	(95.9)	8	(100.0)		48	(98.0)	7	(87.5)	
Yes	2	(4.2)	0	(0.0)	0.92	2	(4.1)	0	(0.0)	0.93	1	(2.0)	1	(12.5)	0.26

Supplemental material 2, Table 1. Association between petroleum and chlorinated solvents and tobacco exposure during pregnancy and non-occupational chemical exposure.

	Mandelic acid					Hippuric acid					Tetrachloroethylene				
	< LOQ		≥ LOQ			< LOQ		≥ LOQ			< LOQ		≥ LOQ		
	N	(%)	N	(%)	p-value	N	(%)	N	(%)	p-value	N	(%)	N	(%)	p-value
**Tobacco consumption during pregnancy**															
No	34	(65.4)	4	(80.0)		28	(77.8)	10	(47.6)		20	(66.7)	18	(66.7)	
Yes	18	(34.6)	1	(20.0)	0.51	8	(22.2)	11	(52.4)	0.04	10	(33.3)	9	(33.3)	0.98
**Passive smoking**															
No	19	(38.8)	1	(25.0)		14	(42.4)	6	(30.0)		13	(48.1)	7	(26.9)	
Yes	30	(61.2)	3	(75.0)	1.00	19	(57.6)	14	(70.0)	0.37	14	(51.9)	19	(73.1)	0.11
missing	3	-	1	-		3	-	1	-		3	-	1	-	
**Damp cleaning**															
< 5 times/month	36	(69.2)	3	(60.0)		23	(63.9)	16	(76.2)		22	(73.3)	17	(63.0)	
between 5 and 20 times/month	13	(25.0)	1	(20.0)		9	(25.0)	5	(23.8)		6	(20.0)	8	(29.6)	
> 20 times/month	3	(5.8)	1	(20.0)	0.47	4	(11.1)	0	(0.0)	0.26	2	(6.7)	2	(7.4)	0.75
**Home renovation activities**															
No	47	(90.4)	5	(100.0)		35	(97.2)	17	(81.0)		29	(96.7)	23	(85.2)	
Yes	5	(9.6)	0	(0.0)	0.95	1	(2.8)	4	(19.0)	0.05	1	(3.3)	4	(14.8)	0.18
**Other chemicals used during leisure activities**															
No	50	(96.1)	5	(100.0)		35	(97.2)	20	(95.2)	0.99	30	(100.0)	25	(92.6)	0.22
Yes	2	(3.9)	0	(0.0)	0.65	1	(2.8)	1	(4.8)		0	(0.0)	2	(7.4)	

Supplemental material 2, Table 1. Association between petroleum and chlorinated solvents and tobacco exposure during pregnancy and non-occupational chemical exposure.

	N	Benzene		Toluene		Trichloroethylene	
		Median [Q1-Q3] ng/g	p value	Median [Q1-Q3] ng/g	p value	Median [Q1-Q3] ng/g	p value
**Tobacco consumption during pregnancy**							
No	38	0.29 [0-1.06]		16.43 [0-155.3]		0.22 [0.10-0.46]	
Yes	19	0.31 [0-1.49]	0.39	4.31 [0-38.23]	0.55	0.18 [0-0.32]	0.41
**Passive smoking**							
No	20	0.08 [0-0.93]		0 [0-58.98]		0.14 [0-0.42]	
Yes	33	0.31 [0-1.22]	0.68	23.36 [0.85-76.79]	0.72	0.25 [0.17-0.39]	0.98
missing	4	-		-		-	
**Damp cleaning**							
< 5 times/month	39	0.29 [0-1.06]		2.66 [0-60.34]		0.20 [0-0.35]	
between 5 and 20 times/month	14	0.40 [0-1.53]		44.10 [14.35-246.0]		0.26 [0.18-0.39]	
> 20 times/month	4	0.69 [0.15-16.56]	0.64	30.04 [0-76.79]	0.24	0.08 [0-0.31]	0.36
**Home renovation activities**							
No	52	0.30 [0-1.08]		4.05 [0-85.85]		0.20 [0-0.34]	
Yes	5	0.23 [0-0.78]	0.65	154.4 [16.66-292.2]	0.23	0.49 [0.33-0.51]	0.02
**Other chemicals used during leisure activities**							
No	55	0.24 [0-1.07]		9.33 [0-98.22]		0.22 [0.11-0.39]	
Yes	2	20.50 [0.29-40.72]	0.31	21.01 [3.79-38.23]	0.8	0.07 [0-0.15]	0.17

LOQ : Limit of quantification
